# Psychometric properties of the Thai Mood and Feelings Questionnaire (MFQ) for adolescent depression

**DOI:** 10.1186/s13034-021-00372-8

**Published:** 2021-04-09

**Authors:** Nanthaka Fuseekul, Faith Orchard, Shirley Reynolds

**Affiliations:** 1 School of Psychology and Clinical Language School Sciences, University of Reading, Reading, RG6 6AL UK; 2 School of Psychology, University of Sussex, Sussex, UK

**Keywords:** Depression, Adolescence, Self-report, Measurement, Psychometric properties

## Abstract

**Background:**

The Mood and Feelings Questionnaire (MFQ) is a widely used screening tool for child and adolescence depression but has not been validated with young people in Thailand. This study aimed to assess the reliability and validity and to determine the optimal clinical cut-off of the Thai MFQ.

**Methods:**

The Thai MFQ was evaluated in two parts. In part 1, The MFQ was translated and back translated into the Thai language and piloted on a small number of Thai adolescents. Then 1275 young people aged 12–18 years from three secondary schools in Thailand completed the MFQ and related measures of depression. In part 2, 138 students were invited to take part in a structured diagnostic interview (the Thai translation of the Kiddie Schedule for Affective Disorders and Schizophrenia for School-Age Children -Present and Lifetime Version (the K-SADS-PL). Of those, 103 students were interviewed and completed the Thai MFQ a second time to assess test–retest reliability. Receiver Operating Characteristics (ROC) analyses were conducted to evaluate diagnosis accuracy and examine the optimal cut-off score of the Thai MFQ.

**Results:**

The Thai MFQ had excellent internal consistency (α = 0.92) and good to moderate test–retest reliability in 2-week and 4-week intervals. The Thai MFQ also had good convergent validity with related measures of depression. The ROC analyses demonstrated that the Thai MFQ also had excellent accuracy distinguishing between depressed and non-depressed adolescents [AUC = 0.95, 95% CI [0.92, 0.99]. A total score of 28 on the Thai MFQ was the optimal cut-off score (sensitivity was 0.97 and specificity was 0.83).

**Discussion:**

The Thai MFQ demonstrated excellent psychometric properties and accurately distinguished between depressed and non-depressed adolescents. It is appropriate to use as a screening measure to identify adolescents with depression in community settings in Thailand.

**Supplementary Information:**

The online version contains supplementary material available at 10.1186/s13034-021-00372-8.

## Introduction

Depression is a common mental health problem in adolescents worldwide [1]. Adolescence is a life stage when individuals are at high risk to the onset of depression [2]. At any point in time approximately 2.6% of young people worldwide are experiencing a depressive disorder [3]. Depression during this period of life is associated with impaired functioning across multiple life areas, including school and education, interpersonal problems, and suicidal behaviour [4]. These impairments have long term effects in the transitions to adulthood [5]. The long-term consequences of experiencing depression adolescence, mean that it is vital that assessment tools for identifying depression can accurately establish the presence of depressive symptoms in order to provide appropriate prevention and treatment.

Many self-report measurement instruments are used to assess depression in adolescents in research and clinical practice [6]. Most of these instruments have been developed for use in the USA or Europe and their psychometric properties have been evaluated with young people from those countries. The validity of these instruments varies when they are translated and used in new contexts and with young people from other cultures [7, 8]. It is crucial to assess the psychometric qualities of translated questionnaires and to establish their validity and reliability when used in different cultural contexts [9, 10].

There are over 87 million people with depression living in South-East Asia [11]. Most of the countries in this region belong to the lower- and upper-middle income countries (LMICs and UMICs) group [12]. Thailand is one of the UMICs in South-East Asia [13]. The Thai Department of Mental Health [14] reported that the point prevalence of major depressive disorder among the Thai population aged 15 and above was 2.4%. Although this prevalence rate is equivalent to the worldwide rate, the country has faced significant mental health challenges for a range of reasons including lack of investment and structural barriers that have limited the development of mental health services for young people [15]. As a result, depression in Thai adolescents is largely unrecognised, undiagnosed and not appropriately treated. To develop better support and services for depressed young people in Thailand, it is important to identify screening tools for depression that are valid and reliable.

The Children’s Depression Inventory (CDI) [16], and the Centre for Epidemiologic Studies-Depression Scale (CES-D) [17] have been used to measure of adolescent depression in Thailand. Based on these questionnaires the prevalence of elevated symptoms of depression in Thai adolescents is highly variable, ranging from 11 to 46.2% [18–22]. In studies that have assessed depression using diagnostic interviews based on Diagnostic and statistical Manual of mental disorders (DSM) criteria rates of depression were much lower and less variable, i.e., in the range from 1.6–7.7% [23, 24]. The discrepancy in point prevalence based on these self-report questionnaires and interviews in Thailand were similar to studies conducted in other countries e.g., China, Uganda, Egypt, and Iran [25–28]. This discrepancy suggests that self-report measures of depression are highly sensitive but lack specificity and require further psychometric testing and improvement.

The measures of adolescent depression that have been used in Thailand have some shortcomings in their ability to screen and measure the severity of depression. For example, in the CDI, each item includes three statements that describe symptoms of varying severity and which a young person is asked to endorse the one that best describes their symptoms. This requirement to select the “best” description is cognitively demanding, and some items have complex wording and may be confusing (e.g., I do not do what I am told most of the time” or “I do not like being with people many times” [6, 29]. The CES-D also has some problems with its construct in particular it does not include all of the symptoms of major depressive disorder, e.g., suicidal ideation [26]. It also includes items that are not symptoms of depression i.e. “People were unfriendly” and “I felt that people dislike me” [30–33]. Given these shortcomings there is potential benefit in identifying an alternative self-report measurement of depression symptoms in Thai young people.

In the UK, the Mood and Feelings Questionnaire (MFQ) [34] is considered to be the ‘gold standard’ screening tool for depression [35]. The MFQ was developed to screen DSM-IIIR depression symptoms in young people aged 8–18 years. It has been validated in clinical and community settings and has high internal consistency and good test–retest reliability [36–38]. The MFQ discriminates between depressed and non-depressed young people [37, 38] The MFQ has been translated into many languages such as Norwegian [39], Arabic [40], and Swedish [41] using the black-translation method. The MFQ is therefore a plausible measure to adapt for a Thai population although the psychometric properties of a Thai version would need to be established.

In addition, to identify the diagnostic accuracy of the MFQ in Thailand it is necessary to compare the results of the MFQ with the gold standard diagnostic tool for depression. The Kiddie Schedule for Affective Disorders and Schizophrenia for School-Age Children -Present and Lifetime Version (K-SADS-PL) [42] is considered to be the gold standard diagnostic instrument for depression in children and young people. The K-SADS-PL has good reliability and shows good convergent validity with self-report rating scales of depression [36, 42, 43]. The K-SADS-PL has also been translated and validated in other countries, for instance, Korea [44] and Iran [45] and shows good psychometric properties on those versions.

The present study had two aims. The first was to translate the MFQ into Thai and to assess its internal reliability, test–retest reliability, construct validity and criterion validity in a large community sample of young people. The second aim was to determine the optimal clinical cut-off of the Thai MFQ when compared against a structured diagnostic interview (the K-SADS-PL), which was also translated into the Thai language. These aims were addressed in two parts of our study.

## Methods

### Ethical approval

This study was approved by University of Reading Research Ethics Committee, the UK (SREC 2018/105) and Chiang Mai University Research Ethic Committee, Thailand (CMUREC 61/073). Informed written consent was obtained from all participants and from the parents of young people under 18 years of age based on standards prescribed by Chiang Mai University Research Ethic Committee.

### Part 1 Psychometric properties of the Thai Mood and Feelings Questionnaire

#### Participants

**Fig. 1:**
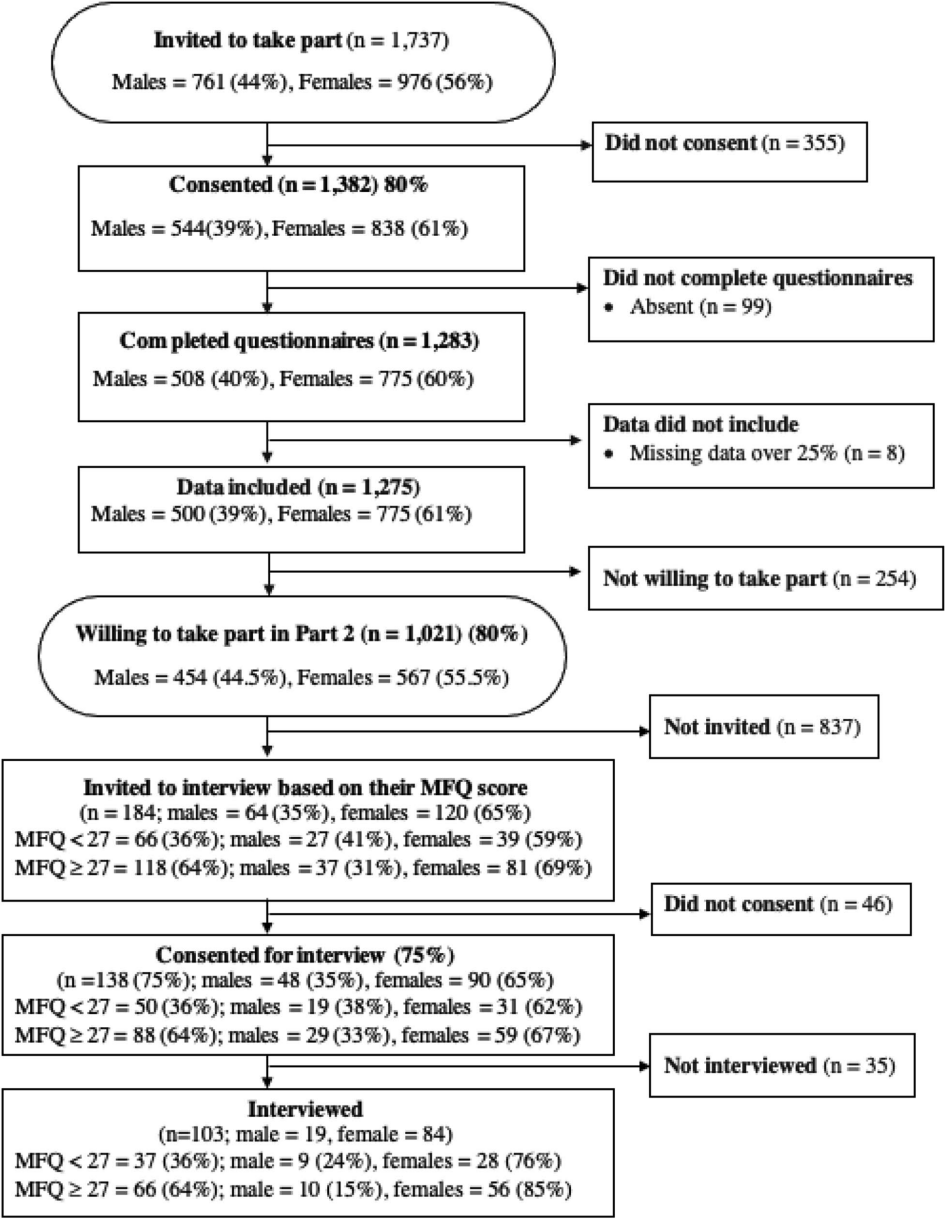
A flowchart of the participants recruitment for Part 1 & Part 2 of the study

Three public secondary schools in Thailand agreed to take part in the study; two from Phayao province and one from Chiang Mai province. A total of 1737 adolescents aged between 12 and 8 years old were invited to take part (male 44%, female 56%). Eighty percent (n = 1382) of those who were invited agreed to take part and provided written consent from their caregiver as well as written assent for themselves. Ninety-nine young people for whom consent was obtained were not included because they were absent from school on the day the research was conducted. Eight young people were excluded because more than 25% of their data were missing. Of the 1275 adolescents who took part 39% were male (n = 500) and 61% (n = 775) were female; significantly more females than males took part, χ^2^(1) = 10.72, *p* = 0.001. The flow chart of participant recruitment is presented in Fig. 1.

#### Measures

*The Mood and Feelings Questionnaire *(*MFQ*) [34] is a 33-item questionnaire used to assess depression symptoms in children and adolescents aged between 8 and 18 years old. Items are rated for the past 2 weeks. Each item is rated on a three-point Likert scale of “true” (“2”), “sometimes true” (“1”) or “not true” (“0”), yielding a maximum score of 66. The MFQ has high internal consistency (α = 0.94) [35] and can discriminate between depressed and non-depressed children and adolescents sampled from both clinical and community settings [37, 38]. A cut-off of 27 provided optimal sensitivity and specificity in identifying young people who met criteria for a diagnosis of major depressive disorder [36]. The MFQ has been adapted and translated into a number of languages and demonstrated an excellent internal consistency in translated versions e.g., Norwegian (α = 0.91) [39], Arabic (α = 0.92) [40], and Swedish version (α = 0.93) [41].

*The Children’s Depression Inventory* (*CDI*) [16] is a 27-item self-report questionnaire that measures of depression for children and adolescents ages 7 to 17 years. It measures current levels of depression symptoms on five factors: negative mood, interpersonal problems, negative self-esteem, ineffectiveness, and anhedonia. The CDI has good internal consistency and test–retest reliability [16]. The CDI has been translated into Thai [46] and had good internal consistency (α = 0.83).

*The Strengths and Difficulties Questionnaire* (*SDQ*) [47] is a 25-item screening questionnaire used to assess mental health in children and adolescents aged 4–16 years old. The SDQ has four subscales to measure emotional symptoms, peer problems, hyperactivity and inattention, and conduct problems and one subscale that assesses prosocial behaviour. The SDQ Thai version [48] has acceptable internal consistency, e.g., total difficulties subscales (α = 0.70) and emotional subscale (α = 0.63).

#### Translation of the MFQ

Permission was obtained from the developer for translation of the MFQ to the Thai language. The translation and adaptation of the MFQ followed guidance cross-cultural adaptation of psychological instruments [49]. First, the original version of the MFQ was translated into Thai by the first author (NF) a native Thai speaker. The Thai version was adapted for linguistic context and aimed to preserve all essential characteristics of the original version. Next, the Thai translation was back translated into English by a bilingual (Thai–English) translator from the Psychology Department at Chiang Mai University who did not have knowledge about the original instrument. The original version of the MFQ was then evaluated and compared with the back-translation. All differences were resolved by discussion. The consensus version was adequately adapted culturally and linguistically for the target population.

The Thai translation of the MFQ was then piloted with five young people in Thailand to check if they interpreted the questions as intended. Adolescents were asked to express their understanding of the measure and to suggest any changes they considered necessary. Adolescents understood all items on the MFQ. Once this was completed, the MFQ was released for use in this study (see Additional file 1).

#### Procedures

In part 1 of the study, following receipt of informed consent, adolescents (N = 1275) were given and completed a pack of questionnaires in their classroom during the school day at a time convenient to their schools. These were distributed and collected by the first author (NF). These data were used to examine internal consistency, descriptive statistics and construct validity of the MFQ. Participants were also asked to indicate if they were willing to take part in Part 2 of the study (diagnostic interview) and, if so, to provide contact details for the researcher.

Research procedures and risk management process were discussed with schools in advance. On the day of data collection, the researcher identified students whose response to the questionnaires suggested that they were at risk of suicide or self-harm. The researcher informed a member of the school safeguarding team on the same day as the risk was disclosed, following school’s safeguarding guidelines.

#### Statistical analysis

Data were analysed using SPSS version 25. Fewer than1% of items had missing values. Participants’ data were excluded from the analysis if more than 25% was missing (n = 8). Where fewer than 25% of item were missing, mean item substitution was used to impute missing data. An independent t-test was used to examine the mean difference between male and female. The association between age and depression symptoms was assessed by a Pearson’s correlation. Internal consistency of the MFQ was assessed with Cronbach’s alpha. Convergent validity of the MFQ was assessed through Pearson correlation coefficients between total score of the MFQ and the CDI total score and the SDQ Emotion symptoms subscale.

### Part 2: Diagnostic interview

#### Participants

One thousand and twenty-one (80%) adolescents agreed to take part in a follow up interview. Based on the MFQ score in Part 1, participants were divided into two groups (elevated, i.e., 27 and above, and sub-threshold, i.e., below 27 [36]). Random samples were then selected from each group. MadCalc version 19.7 was used to estimate a required sample size for a Receiver Operating Characteristics (ROC) curve. The power analysis determined a minimum sample size of 31 participants in each group (i.e. elevated and sub-threshold group) included in ROC analyses to achieve a sufficient power of 0.80 with an Area Under the Curve (AUC) of 0.70 and α = 0.5. To ensure that there were sufficient participants with elevated symptoms of depression, young people with elevated MFQ scores were over-sampled in a ratio of 1.8 (elevated) to 1.0 (sub-threshold). Of the 184 young people invited to the interview, 138 young people and their guardians consented to take part (75%). Twenty-five young people could not subsequently be contacted, and ten young people were not at school on the day of the interview. The remaining 103 young people were interviewed (see Fig. 1). Based on their MFQ scores on the second administration participants were classified as having an elevated Thai MFQ (n = 44) or sub-threshold Thai MFQ (n = 59).

#### Measure

*The Kiddie Schedule for Affective Disorders and Schizophrenia for School-Age Children; Present and Lifetime Version; Depressive disorder *(*K-SADS-PL DSM-5*) [50] is a semi-structured interview with well-established psychometric properties that generates a reliable and valid diagnosis of depression in children and adolescents [42]. The K-SADS-PL has been widely used in epidemiological and treatment research [45, 51] and has also been translated and adapted into many languages [44, 45, 52] In the present study, the depressive disorder section of the K-SADS-PL, revised to be compatible with DSM-5 diagnoses, was used to determine the presence/absence of depressive disorder in Thai adolescents.

#### Translation of the K-SADS-PL; Depressive Disorder

The translation and adaptation of the K-SADS-PL followed the procedure in Part 1. The K-SADS-PL; Depressive disorder was translated into Thai by the first author (NF). The Thai version was adapted for linguistic context and aimed to preserve all essential characteristics of the original version. The K-SADS-PL; Depressive disorder Thai version was back-translated into English by a bilingual child and adolescent psychologist (Thai-English) translator from the Psychology Department at Chiang Mai University. The back-translation version was reviewed and compared with the original version. The final translation was fixed by consensus. The completed Thai version of the K-SADS-PL; Depressive disorder was administered to five young people in Thailand to check if they understood the questions of the measure. Once this was completed, the K-SADS-PL was used in this study.

#### Procedures

Part 2 of the study took place between 11 and 30 days after participants completed the self-report questionnaires. Participants took part in the K-SADS-PL interviews, which were conducted in a quiet room at school by the first author (NF), and completed the MFQ after the interviews. The interviews were audio-taped and detailed assessment notes were taken. Subsequently each interview recoding (n = 103) was coded according to K-SADS-PL diagnostic criteria by the first author (NF). NF has enhanced DBS and was trained to deliver and score the K-SADS-PL through training which included verbal instruction, watching training videos, and participating in diagnostic consensus supervision meetings where each individual symptom was discussed for parent and child, and a consensus agreed for each symptom before reaching an overall diagnosis decision. To check reliability of the diagnosis, 10% of the samples were double-rated by an experienced K-SADS-PL assessor (FO). Inter-rater reliability for the presence of depression diagnoses on the K-SADS-PL was κ = 0.80, and on individual symptoms was κ = 0.75.

The researcher discussed the diagnostic interview and safeguarding procedures with the schools before the interview. The school’s safeguarding guidelines were followed on the day of interview. Therefore, the researcher alerted the school safeguarding team on the same day as the risk was disclosed (i.e., experienced or thoughts of self-harm or suicide). Research procedures and safeguarding processes were also explained in information sheets to students and their parents.

#### Statistical analysis

In Part 2, the MFQ data from the second administration were used and analysed using SPSS version 25. Diagnoses were assigned according to the K-SADS-PL. There was no missing data on the MFQ or demographic information for the sub-sample of 103. Chi-square test was used to determine association of categorical variables. Mean differences of the MFQ were examined for young people who were given a diagnosis of MDD versus those who did not receive a diagnosis of MDD. Test–retest reliability was assessed by using Intraclass correlation coefficients (ICCs) absolute agreement model [53]. Because the interval between completing the MFQ at first and second administration varied, participants were allocated to two groups. Group one completed the MFQ approximately 2 weeks apart (11–19 days, n = 40); group two completed the MFQ approximately 4 weeks apart (20—30 days, n = 63). Receiver Operating Characteristic (ROC) analyses [54] were conducted to examine the ability of the MFQ to discriminate between individuals with and without a diagnosis of Major Depressive Disorder.

The ROC curve analysis provided information regarding sensitivity (the probability that the test correctly classifies subject with condition as positive), specificity (the probability that the test correctly classifies subject without condition as negative), positive predictive value (the probability of the presence of disease in those with a positive test result), and negative predictive values (the probability of the absence of disease in those with a negative test results) [55] of the MFQ for candidate cut-off points. As the MFQ is designed to be used primarily as a screening instrument, the optimal cut-off was determined by favouring sensitivity over specificity; therefore, minimum sensitivity was set as 80% as minimum specificity was set at 70% [56]. The accuracy of the MFQ in detecting depression was evaluated by the area under the curves (AUC). AUC measures the ability of screening measures to correctly classify those with and without the disease. AUC of 1.0 represents perfect diagnostic accuracy, AUC greater than 0.9 reflects high accuracy, 0.7–0.9 moderate accuracy and below 0.7 indicates low diagnostic accuracy [54].

## Results

### Part 1: Psychometric properties of the Thai MFQ

#### Demographic characteristics

All adolescents described themselves as Thai national. Their mean age was 15.04 years (SD = 1.73; range = 12–18). Significantly more females than males took part in the study (females = 60.8%). There was no statistically significant difference in age between males and females (see Table 1).

**Table 1:** Demographic and descriptive statistics of the MFQ

Characteristics	Mean (SD)	Test
Demographics		
Age: 12–18 years	15.04 (1.73)	*t*(1273) = − 1.62, *p* = .76
Males: n = 500	14.95 (1.73)	χ^2^(1) = 10.72, *p* = .001
Females: n = 775	15.11 (1.73)	
MFQ total: ranged 0–55	14.65 (9.97)	*t*(1273) = − 3.8, *p* < .001, *d* = .22
Male	13.32 (8.62)	
Female	15.51 (10.66)	

MFQ:  Mood and Feeling Questionnaire; SD: standard deviation

#### Descriptive statistics of the MFQ

The mean MFQ score for the whole sample was 14.65 (SD = 9.97). Females reported MFQ scores that were significantly higher than males (see Table 1). There was no association between age and the MFQ score, *r* = 0.01 *(p* = 0.67*)*. MFQ items that were most highly endorsed were “I felt miserable or unhappy” (M = 0.98, SD = 0.56), “I ate more than usual” (M = 0.90, SD = 0.60), and “It was hard for me to make up my mind” (M = 0.87, SD = 0.61).

### Reliability

#### Internal consistency of the MFQ

The internal consistency of the Thai MFQ was excellent, Cronbach’s alpha α = 0.92 [57]. The corrected item-total correlation coefficients showed that each item contributed substantially to the total MFQ score; these ranged from *r* = 0.32 to *r* = 0.66; except for item 4 with a correlation *r* = 0.20: “I ate more than usual”. The alpha value of the scale after deletion of item 4 did not change (α = 0.92). Therefore, all items were retained.

### Validity

#### Construct validity

To assess the convergent validity of the Thai MFQ we examined correlations between the MFQ total score, total scores on the CDI, and the SDQ emotional symptoms subscale. There was a strong positive correlation between the MFQ and the CDI (*r*  = 0.76, n = 1275, *p* < 0.001), and between the SDQ emotional symptoms subscale (*r*  = 0.71, n = 1275, *p* < 0.001) [58].

### Part 2: Establishing cut-off for the Thai MFQ

At the second administration of the MFQ, the mean score of the elevated MFQ group (n = 44) was 36.07 (*SD* = 6.95) and the mean MFQ for the sub-threshold group (n = 59) was 15.29 (*SD* = 6.06), *t*(101) = − 16.17, *p* < 0.001. There was no significant age difference between participants with elevated MFQ scores and those with sub-threshold scores *t*(101) = − 1.31, *p* = 0.11. There were more females than males in both groups; elevated MFQ, χ^2^ (1) = 32.06, *p* < 0.001 and sub-threshold MFQ χ^2^ (1) = 10.52, *p* < 0.01 (see Fig. 1).

### Test–retest reliability

Test–retest reliability of the MFQ was assessed by examining the intraclass correlation coefficient of the MFQ. Forty participants completed the MFQ 2 weeks apart and 63 completed the MFQ 4 weeks apart. Two-week test–retest was reliability was good; ICC = 0.88, *p* < 0.001), 95% CI [0.76, 0.94] and 4-week test–retest was moderately reliable; ICC = 0.57, *p* < 0.001), 95% CI [0.13, 0.78] [53].

### Receiver Operating Characteristic of the MFQ

ROC curve analysis was performed to assess the accuracy of the MFQ to discriminate between depressed and non-depressed adolescents. The diagnostic accuracy of the MFQ was high [AUC = 0.95, 95% CI [0.92, 0.99] [54]. The MFQ accurately classified young people as depressed or non-depressed. Table 2 shows the predicted percentage of sensitivity, specificity, positive predictive value, and negative predictive value for each MFQ score. The examination of the ROC curve suggested that the optimal cut-off point for screening depression was 28, where the corresponding sensitivity (0.97) and specificity (0.83) most closely intersected.

**Table 2:** Sensitivity, specificity, positive predictive value, and negative predictive value for different cut-off points of the MFQ

Cut-off (n = 103)	Sensitivity	Specificity	PPV	NPV
25	0.97	0.76	0.64	0.98
26	0.97	0.79	0.67	0.98
27	0.97	0.81	0.68	0.98
28*	0.97	0.83	0.71	0.98
29	0.93	0.88	0.76	0.97
30	0.90	0.90	0.80	0.96

^*^Optimal cut‐off value

PPV: Positive predictive value; NPV: Negative predictive value

### Criterion validity

Of the sub-sample of 103 adolescents who had the K-SADS-PL diagnostic interview, 31 met criteria for major depressive disorder and 72 did not meet the criteria. Adolescents who met diagnostic criteria for major depression reported significantly higher MFQ score (*M* = 37.68, SD = 7.32) than those who did not meet criteria (*M* = 18.35, SD = 8.71), *t*(101) = − 10.82, *p* < 0.001, *d* = 2.4. Using the MFQ cut-off of 28 (the present study cut-off), the majority of participants were identified correctly as depressed or non-depressed (n = 90, 87.38%) and 13 participants had a discrepant diagnostic status (see Table 3).

**Table 3:** Frequencies for depression determination using the MFQ at cut-off of 28 and the K-SADS-PL diagnosis

MFQ scores	K-SADS-PL diagnosis		Total	*p*
	Depressed	Non-depressed		
Elevated (MFQ ≥ 28)	30	12	42	χ^2^ (1) = 57.58, *p* < 0.001
Sub-threshold (MFQ < 28)	1	60	61	
Total	31	72	103	

MFQ: Mood and Feeling Questionnaire; K-SADS-PL: The Kiddie Schedule for Affective Disorders and Schizophrenia for School-Age Children; Present and Lifetime Version; Depressive disorder

## Discussion

This is the first study to evaluate the psychometric properties and validity of the Thai MFQ in a community sample of young people in Thailand. This study also determined the optimal clinical cut-off of the Thai MFQ when compared against a structured diagnostic interview (the K-SADS-PL). The results indicate that the Thai MFQ is a reliable and valid instrument for assessing depression in Thai adolescents. The Thai MFQ had excellent internal consistency, comparable to the original English language version in the previous studies [36, 38] and other versions of the scale such as Norwegian [39], Arabic [40], and Swedish versions [41]. Regarding validity, the Thai MFQ reported high correlation with scales that measure depressive symptoms as well as good levels of sensitivity and specificity. The Thai MFQ also had good construct validity and good to moderate test–retest reliability in two-week and four-week intervals.

The Thai MFQ had excellent accuracy distinguishing between depressed and non-depressed adolescents. The AUC was better than those reported found in the previous validation studies in the original English language version [36–38] and translated version [39, 41]. The cut-off point that best combined sensitivity and specificity corresponds to a score of 28. This is slightly higher than the cut-off of 27 proposed by Wood et al. [36] in the UK, and for other translations of the scale (e.g., Arabic and Norwegian). This cut-off is comparable to that found in another study exploring the psychometric properties of the English language MFQ in a psychiatric sample of young people in New Zealand [59].

The psychometric qualities of the Thai MFQ were excellent. Furthermore, adolescents found the phrasing of the items and structure of the Thai MFQ easy to understand and to follow, whereas some young people, especially younger adolescents, had difficulty understanding some items of the CDI and asked for clarification, e.g. “I do not do what I am told most of the time”.

One strength of this study is the large community sample with a high response rate. The results of this study are therefore likely to be representative of the Thai adolescent population. This study also examined a range of psychometric properties of the Thai MFQ and used the gold standard structured clinical interview, the K-SADS-PL to discriminate between participants with and without depression. This procedure provided unbiased estimate of the diagnostic accuracy of the Thai MFQ. However, some limitations mean that our findings should be considered cautiously. First, this study recruited young people from the community, therefore the results of our study may not generalise to young people recruited in other settings, e.g., primary care and mental health settings, where the prevalence of depression is expected to be higher. Future research should aim to validate a Thai MFQ with a clinical sample. In addition, the diagnostic interviews were administered and coded by one researcher, which could introduce bias. We mitigated this by randomly checking the coding of the K-SADS-PL interviews with as experienced K-SADS assessor and trainer (FO). Future validation of the Thai MFQ should include multiple diagnostic interviewers and a comprehensive assessment of inter-rater reliability. Further, despite good psychometric properties of the Thai MFQ, this is not sufficient to establish cross-cultural measurement invariance across cultures. This should also be incorporated into future studies.

Despite these limitations the psychometric properties of the Thai MFQ, its coverage of all depression symptoms, and ease of administration and scoring, suggest that the Thai MFQ may be the most efficient measure of detecting adolescent depression in community settings. The Thai MFQ could also be used with individuals attending primary care or mental health services. In addition, the cut-off score indicated in this study could help identify young people who are most likely to have an episode of major depressive disorder. Using the Thai MFQ could therefore improve the efficiency of identifying adolescents with depression in Thailand. The Thai MFQ could also help mental health staff in community settings, such as school psychologists or guidance counsellors, to effectively screen young people for depression and to determine whether further assessment and referral are indicated. The Thai MFQ would potentially help save time and resources in a country where mental health resources for young people are limited [15].

## Conclusion

Our findings suggest that the Thai MFQ is reliable, valid, and easily understood by young people. We provide promising evidence that the Thai MFQ is an appropriate screening measure for depression in Thai adolescents and may also be suitable for use in primary care and mental health settings, but this should be established before widespread use. The Thai MFQ could fill a gap in the assessment tools available to researchers and clinicians for assessing depression in Thai young people. The findings also provide support to the existent literature for the MFQ validation across cultures. However, the equivalence of the Thai MFQ across different cultures has not yet been examined. Therefore, evaluating the measurement equivalence of the MFQ across cultures should be examined in future studies.

## Supplementary Information

**Additional file 1.** The Thai MFQ. The Thai Mood and Feelings Questionnaire (MFQ).

## Data Availability

The Thai Mood and Feelings Questionnaire (MFQ) is included within the article. The datasets and other materials used during the current study are available from the corresponding author on reasonable request.

## Abbreviations

AUC: Area under the curves
MFQ: Mood and Feelings Questionnaire
CDI: The Children’s Depression Inventory
DSM: Diagnostic and Statistical Manual of Mental disorders
SDQ: The Strengths and Difficulties Questionnaire
K-SADS-PL: The Kiddie Schedule for Affective Disorders and Schizophrenia for School-Age Children; Present and Lifetime Version
ROC: Receiver Operating Characteristic
PPV: Positive predictive value
NPV: Negative predictive value
