# Supplementary material for: Psychometric properties of the Thai Mood and Feelings Questionnaire (MFQ) for adolescent depression
Source: Child Adolesc Psychiatry Ment Health. 2021 Apr 9;15:17. doi: 10.1186/s13034-021-00372-8 (PMC8035723; doi:10.1186/s13034-021-00372-8)
Supplement: Supplementary file 1 — Additional file 1. The Thai MFQ. The Thai Mood and Feelings Questionnaire (MFQ). [file 13034_2021_372_MOESM1_ESM.pdf]

Mood and Feelings Questionnaire (MFQ)

แบบประเมินนี้เป็นแบบประเมินเกี่ยวกับความรู้สึกหรือการกระทำ**ในปัจจุบัน**

กรุณาใส่เครื่องหมาย ✓ ลงในช่องที่ตรงกับความรู้สึกหรือการกระทำของท่านใน**ระยะ 2 สัปดาห์ที่ผ่านมา**

ถ้าข้อความไหนไม่ตรงกับตัวท่าน ให้ทำเครื่องหมาย ✓ ตรงช่อง ไม่จริง

ถ้าข้อความไหนตรงกับตัวท่านบางครั้ง ให้ทำเครื่องหมาย ✓ ตรงช่อง จริงบางครั้ง

ถ้าข้อความไหนตรงกับตัวท่านเป็นส่วนใหญ่ ให้ทำเครื่องหมาย ✓ ตรงช่อง จริง

กรุณาใส่เครื่องหมาย ✓ ในทุกข้อความ	ไม่จริง	จริงบางครั้ง	จริง
1. ฉันรู้สึกเศร้าหรือไม่มีความสุข			
2. ฉันไม่เพลิดเพลินกับสิ่งใดเลย			
3. ฉันหิวน้อยลงกว่าปกติ			
4. ฉันกินมากขึ้นกว่าปกติ			
5. ฉันรู้สึกอ่อนเพลีย ฉันมักนั่งเฉยๆและไม่ทำอะไร			
6. ฉันเคลื่อนไหวช้าและเดินช้ากว่าปกติ			
7. ฉันกระสับกระส่ายอย่างมาก			
8. ฉันรู้สึกว่าฉันไม่มีอะไรดีเลย			
9. ฉันตำหนิตัวเองในสิ่งที่ไม่ใช่ความผิดของฉัน			
10. มันยากสำหรับฉันที่จะทำการตัดสินใจในเรื่องต่างๆ			
11. ฉันรู้สึกหงุดหงิดและฉุนเฉียวกับพ่อแม่			
12. ฉันรู้สึกว่าฉันพูดน้อยลงกว่าปกติ			
13. ฉันพูดช้าลงกว่าปกติ			
14. ฉันร้องไห้บ่อยมาก			
15. ฉันคิดว่าในอนาคตไม่มีสิ่งดีๆรอฉันอยู่			

กรุณาใส่เครื่องหมาย ✓ ในทุกข้อความ	ไม่จริง	จริงบางครั้ง	จริง
16. ฉันคิดว่าชีวิตฉันไม่มีค่าพอที่จะมีชีวิตอยู่			
17. ฉันคิดถึงความตายหรือภาวะใกล้ตาย			
18. ฉันคิดว่าครอบครัวคงจะดีกว่านี้ถ้าไม่มีฉันอยู่			
19. ฉันมีความคิดเกี่ยวกับการฆ่าตัวตาย			
20. ฉันไม่อยากเจอเพื่อน ๆ			
21. ฉันพบว่าการใช้ความคิดหรือการมีสมาธิเป็นเรื่องยาก			
22. ฉันคิดว่าสิ่งร้ายๆอาจจะเกิดขึ้นกับฉัน			
23. ฉันเกลียดตัวเอง			
24. ฉันคิดว่าฉันเป็นคนไม่ดี			
25. ฉันคิดว่าฉันหน้าตาน่าเกลียด			
26. ฉันกังวลเกี่ยวกับการเจ็บปวด			
27. ฉันรู้สึกโดดเดี่ยว			
28. ฉันคิดว่าไม่มีใครรักฉันอย่างแท้จริง			
29. ฉันไม่สนุกเวลาที่อยู่โรงเรียน			
30. ฉันคิดว่าฉันไม่สามารถดีได้เท่ากับเด็กคนอื่น			
31. ฉันทำทุกอย่างผิดเสมอ			
32. ฉันนอนไม่ค่อยหลับเมื่อเทียบกับปกติ			
33. ฉันนอนมากกว่าปกติ			

วิธีการใช้เครื่องมือ

แบบประเมิน MFQ ใช้กับเด็กและวัยรุ่นอายุ 8 – 18 ปี

วิธีการคิดคะแนน

แบบประเมิน MFQ ประกอบไปด้วยคำถามที่เกี่ยวข้องกับความรู้สึกหรือการกระทำในปัจจุบัน จำนวน 33 ข้อคำถาม ผู้ทำแบบประเมินจะต้องเลือกความถี่ของอาการที่เกิดขึ้นในช่วง 2 สัปดาห์ที่ผ่านมา โดยการให้คะแนนจะให้ตามระดับความถี่ของอาการที่เกิดขึ้น ประกอบด้วย

ไม่จริง = 0

จริงบางครั้ง = 1

จริง = 2

การแปลผล

ผู้ที่ได้คะแนนรวมสูงกว่า 28 คะแนนขึ้นไปจากการคัดกรองถือว่ามีความเสี่ยงในการมีภาวะซึมเศร้า

ชื่อผู้พัฒนา นางสาวนันทกา ฟูสีกุล

ตำแหน่งปัจจุบัน อาจารย์ประจำสาขาจิตวิทยาการศึกษาและการแนะแนว

สถานที่ทำงาน ภาควิชาพื้นฐานและการพัฒนาการศึกษา คณะศึกษาศาสตร์ มหาวิทยาลัยเชียงใหม่

เอกสารอ้างอิง

Angold A, Costello E. J, Pickles A. & Winder F. The development of a questionnaire for use in epidemiological studies of depression in children and adolescents. London Med Res Counc Child Psychiatry Unit. 1987.
